# Faecal Microbiota of Dogs Offered a Vegetarian Diet with or without the Supplementation of Feather Meal and either Cornmeal, Rye or Fermented Rye: A Preliminary Study

**DOI:** 10.3390/microorganisms8091363

**Published:** 2020-09-06

**Authors:** Julia Hankel, Amr Abd El-Wahab, Richard Grone, Birgit Keller, Eric Galvez, Till Strowig, Christian Visscher

**Affiliations:** 1 Institute for Animal Nutrition, University of Veterinary Medicine Hannover, Foundation, 30173 Hannover, Germany; richard.grone@gmx.de (R.G.); birgit.keller@tiho-hannover.de (B.K.); christian.visscher@tiho-hannover.de (C.V.); 2 Department of Nutrition and Nutritional Deficiency Diseases, Faculty of Veterinary Medicine, Mansoura University, Mansoura 35516, Egypt; amrwahab5@mans.edu.eg; 3 Helmholtz Center for Infection Research, Inhoffenstraße 7, 38124 Braunschweig, Germany; Eric.Galvez@helmholtz-hzi.de (E.G.); Till.Strowig@helmholtz-hzi.de (T.S.); 4 Hannover Medical School, Carl-Neuberg-Straße 1, 30625 Hannover, Germany

**Keywords:** dog, gut bacteria, *Firmicutes*, *Bacteroidetes*, 16S rRNA gene, novel nutritional trends, fermentation, fibre

## Abstract

Anthropomorphism of dogs has affected feeding and the choice of components present in diets for dogs. Conflicting trends are present: raw or vegetarian appear more prevalent. Animal-derived proteins seem to have unfavourable impacts on intestinal microflora by decreasing the presence of *Bacteroidetes*. This preliminary study evaluates whether effects of diets with animal proteins on intestinal microbiota can be compensated by the addition of certain carbohydrates to dog diet. Eight female beagles were included in a cross-over study and fed a vegetarian diet or the same diet supplemented with feather meal (2.7%) and either 20% of cornmeal, fermented or non-fermented rye (moisture content of the diets about 42%). A 16S rRNA gene amplification was performed within the hypervariable region V4 on faecal samples and sequenced with the Illumina MiSeq platform. The *Firmicutes*/*Bacteroidetes* ratio tended to shift to the advantage of *Firmicutes* when feather meal and cornmeal were added (*Firmicutes*/*Bacteroidetes* ratio of 5.12 compared to 2.47 when offered the vegetarian diet) and tended to switch back to the advantage of *Bacteroidetes* if rye: fermented (2.17) or not (1.03) was added. The addition of rye might have the potential to compensate possible unfavourable effects of diets with animal proteins on intestinal microbiota of dogs.

## 1. Introduction

Not only the canine genome but also the bacterial population harboured by the canine gut seem to be shaped during domestication of dogs from wolves to adapt to dietary changes induced by cohabitation with humans [1]. Even if the dog is a carnivorous species, the basic ingredients for most commercial diets for dogs are cereal grains as a carbohydrate source. These ingredients supply energy and fibre and could represent 30–60% of the dry matter in dry foods for dogs [2,3]. Nowadays, anthropomorphism of dogs has resulted in pet owner preference for pet foods containing ingredients known from their own diets [4]. People who avoid eating animals often feed or are interested in feeding plant-based or vegan diets to their pets [5]. Feeding dogs unconventional diets such as vegetarian, raw and homemade diets appears to be more prevalent [6]. Raw meat-based diets often include higher amounts of protein and fat compared to commercial foods, with relatively low amounts of total carbohydrate and dietary fibre, showing the risk of developing adverse effects like increased colonic fermentation and gas production [7]. The administration of a strict vegan or vegetarian diet results in a significant shift in the microbiota of humans [8,9] with certain benefits like having a greater richness compared to omnivores [9] or reducing intestinal inflammation [10]. Nevertheless, Losasso et al. [9] expected higher dissimilarity in bacterial community composition between vegans, vegetarians and omnivorous humans; the high similarity between the different eating habits is probably due to the common nutrient intakes rather than divergent foods. In dogs, it could be shown that unprocessed protein-rich diets based on low-quality animal sources have an impact on the physicochemical characteristics of the intestinal chyme (higher faecal water content and a less well-formed stool) compared to that of dogs fed extruded dry diets with higher carbohydrate level [11]. In addition, impacts were also seen on the microbial metabolic activity (indicated by a more alkaline faecal pH) as well as on the composition of the intestinal microflora (higher faecal counts of *Clostridium perfringens* and antagonistic effects on bifidobacteria) [11]. Furthermore, the microbiota of dogs fed diets enriched in fibres and carbohydrates are more complex and showed a lower abundance of *Fusobacteria* and *Actinobacteria* and higher abundance of *Bacteroidetes* compared to that of dogs fed a diet based on a high amount of animal proteins and fats [1]. An observed reduction of the butyrogenic bacterium *Faecalibacterium* spp. in faecal samples of dogs fed a diet based on a high amount of animal proteins and fats, which is associated with a healthy microbiota in humans [12] and anti-inflammatory features [13], led the authors to the assumption that a meat-based diet is less protective against inflammatory activity [1]. With these observations as a basis, the impression could be created that for a healthy intestinal microbiome, a vegetarian diet of a carnivorous dog might be more advantageous, whereby the avoidance of protein-rich diets of poor protein quality also seems to play a role. However, taken the carnivorous physiology of dogs into account, the suitability of plant-based diets in meeting the nutritional requirements has been questioned, with regard to different amino acid profiles and potential reduced nutrient availability due to anti-nutritive factors and non-starch polysaccharides in plants [14]. Most animal sources are rich in lysine and tryptophan, while lysine is often the first limiting amino acid in cereal-based dog foods [15]. The sustainability of food animal production is greatly enhanced by recycling animal by-products like feather meal during the rendering process and using those by-products as feed ingredients for companion animals [16]. Even without having the highest lysine contents under the animal-derived by-products, lysine contents are higher compared to most plant-based ingredients [17]. Apparent protein digestibility of dogs was not affected when diets containing 5 to 10% hydrolysed feather meal were offered but faecal quality was impaired, whereby the author suspected influences on the microbiota behind this observation [18].

The aim of the present study was to investigate whether certain additives to a non-vegetarian diet have effects on the faecal microbiota similar to those seen when feeding a vegetarian diet. In other words, the question should be clarified whether the supposed unfavourable effects of an animal-derived protein in diets for dogs on faecal microbiota can be compensated by the addition of certain carbohydrates. Maize is a common component and carbohydrate source that can be found in food for dogs. The contained resistant starch [19] is only poorly broken down by the host’s own enzymes and passes through the small intestine relatively unharmed [20] before it is also fed to microbes for degradation in the large intestine [21]. Rye, popular in human nutrition, shows the highest fermentation rate and extent as well as high butyric acid formation in an in vitro colon model compared to wheat and oat, probably due to its high fructan and water-extractable arabinoxylan content [22]. Finally, fermentation of feed is of special interest because of its potential to have probiotic and prebiotic-like impacts on intestinal and faecal bacterial composition, as already observed in pigs [23].

## 2. Materials and Methods

The study design was examined by the animal welfare officer of the University of Veterinary Medicine Hannover.

### 2.1. Study Design

In a preliminary study, four groups of about three years old healthy female beagle dogs (*N* = 8) of the Institute for Animal Nutrition, University of Veterinary Medicine Hannover, were included in a cross-over study for two months. Dogs had a mean body weight of 10.96 ± 1.31 kg at the beginning of the study. The experiment followed a completely randomized design in a 4 × 1 factorial arrangement of treatment. In each period, four animals received the four different diets for 10 days, and diets were alternated in the following period such that all dogs received all diets once at the trials’ end. The dogs were fed once per day at the same time. About 0.2 g of recently defecated fresh faeces collected on the ninth day were used for microbiota analysis. The obtained 32 faecal samples were stored at −80 °C until simultaneous analysis. The experiment ran without complications. The dogs readily consumed all diets and no signs of dietary intolerance were observed.

### 2.2. Diets

A basic diet was commercially produced as a vegetarian diet and consisted mainly of wheat, broken rice, wheat gluten, rice protein, linseed, sunflower oil and dried peet pulp (Table 1). Three further non-vegetarian diets were created by the supplementation of feather meal to the basic diet (4% on dry matter basis). These non-vegetarian diets were additionally supplemented (30% on dry matter basis) by either rye flour (basic diet + rye), fermented rye (basic diet + fermentate) or cornmeal (basic diet + cornmeal). Additionally, 120 mL of warm water were added to all the diets (except basic diet + fermentate) to have almost identical consistency between the diets.

The rye was fermented by mini-fermenters (Mini-Fermenter 125 L, WEDA Dammann & Westerkamp GmbH, Germany). Briefly, the mini-fermenter was closed after filling and the liquid feed was stirred therein every hour for 60 sec at 900 rotations. A temperature during fermentation of 35–38 °C was ensured over the entire fermentation period (24 h). A rye to water ratio of 1:3 was used in the mini-fermenter. To avoid malfermentation, a freeze-dried, granulated starter culture (Schaumalac Feed Protect XP G, H. Wilhelm Schaumann, Germany), consisting of 1k2079 *Lactobacillus plantarum*, 1k2103 *Pediococcus pentosaceus* and 1k2082 *Lactococcus lactis* was added at the beginning of each fermentation process in a dosage of 2 × 10^5^ cfu/g ingredient.

All diets were analysed for moisture, crude ash, crude protein, crude fat and fibre according to VDLUFA methods [24]. The moisture content was determined by drying to the weight constancy at 103 °C, while the crude ash was analysed by means of incineration in the muffle furnace for six hours at 600 °C. The total nitrogen content was determined by elemental analyser (Elementar, Hanau, Germany), which operates according to the DUMAS combustion method. The crude fat content was determined after acid digestion in the soxhlet apparatus and the content of crude fibre was determined after washing in dilute acids and alkalis. Starch determination was measured polarimetrically (Polatronic E, Schmidt und Haensch GmbH & Co., Berlin, Germany), while the sugar content was analysed in accordance with Luff-Schoorl method by titration with sodium thiosulphate. The mineral content was carried out by atomic absorption spectrometry (Unicam Solaar 116, Thermo, Dreieich, Germany). Contents of lysine, methionine, threonine and tryptophan in the basic diet were calculated with the help of BESTMIX^®^ (adifo software, Maldegem, Belgium) based on values determined by internal analyses of the animal feed producer. For the calculation of lysine, methionine, threonine and tryptophan contents in the non-vegetarian diets, values taken from Rodehutscord et al. [25] for rye and corn as well as from Schulten [26] and Adejumo et al. [27] for feather meal were used. In case of the fermented rye, the same values were taken for the calculation as for rye, because previous analyses have shown that amino acid contents did not differ outside the scope of analysis after the fermentation process [28].

The analysed chemical composition of the diets is shown in Table 2. The diets had a similar water content and are comparable in terms of protein and energy content.

The amount of diet provided was individually determined using standard equations for the daily energy requirements of kennel dogs (0.45 MJ ME/kg body weight^0.75^/d). Metabolizable energy (ME) contents of the diets were estimated based on their chemical compositions, in accordance with the NRC [29].

### 2.3. 16S rRNA Gene Analyses

Until simultaneous analysis, the obtained faecal samples were stored at −80 °C. Afterwards, 16S rRNA gene analyses were done as already described in Hankel et al. [30]. A mixer mill (Retsch MM 400, Haan, Germany) was used to homogenize the chyme before the DNA was extracted based on the DNeasy Blood&Tissue Kit (Qiagen, Hilden, Germany) on an automated liquid handler (Microlab Star, Hamilton Germany GmbH, Gräfelfing, Germany). An additional purification step (Kit: BS 365, BioBasic, Ontario, Canada) was performed before the hypervariable region V4 of the 16S rRNA gene was amplified using the primer F515/R806. Sequencing the amplicons was done on the Illumina MiSeq platform (PE250) and the Usearch8.1 software package (http://www.drive5.com/usearch/) was used to assemble, control quality and cluster obtained reads [31]. Reads were merged with -fastq_mergepairs with fastq_maxdiffs 30. Chimeric sequences were identified and removed using cluster_otus (-otu_radius_pct 3) and the Uchime command included in the Usearch8.1 workflow. Quality filtering was set up with fastq_filter (-fastq_maxee 1); minimum read length, 200 bp. Reads were clustered into 97% ID operational taxonomic units (OTUs). The OTU clusters and representative sequences were determined using the UPARSE algorithm [31]. Taxonomy assignment was done with the help of Silva database v128 [32] and the Naïve Bayesian Classifier from the Ribosomal Database Project (RDP) [33] with a bootstrap confidence cutoff of 70%.

### 2.4. Statistical Analyses

Statistical evaluation of microbiota was performed using R (version 3.5, www.r-project.org) with the R package phyloseq (version 1.24.4) [34]. Ordination was performed using Bray–Curtis dissimilarity-based principal coordinate analysis (PCoA) provided in the R package phyloseq. Permutational multivariate analysis of variance (PERMANOVA) on Bray–Curtis distances was used to quantify the contribution of the factor dietary treatment to the differences in microbial composition of the samples. Sample diversity was measured with the species richness estimators Observed Species, Chao 1 and Shannon index. Comparisons of sample diversity indices between dietary treatments were done using the Kruskal–Wallis test. 

After normalizing the counts, multiple testing included in the R package phyloseq were used to identify taxa with significantly different abundance between dogs offered the basic diet and the basic diet supplemented with either cornmeal, fermented or non-fermented rye. *P*-values were adjusted by the Benjamini and Hochberg (BH) method to control for the false discovery rate (FDR) of 5%. To find OTUs with significantly different abundance between animals fed the basic diet to the other dietary treatments, abundance counts were compared using the R package DESeq2 (version 1.22.2) which uses tests based on the negative binomial distribution [35]. OTUs were filtered using a false discovery rate (FDR) cutoff of 0.01.

## 3. Results

Bray–Curtis dissimilarity-based principal coordinate analysis (PCoA) revealed that four of the 32 analysed samples, belonging to one run, differed from the whole dataset and were therefore excluded from further statistical analyses. The dataset of 28 samples contained 552,201 reads (mean number of reads: 19,721; range: 3,376–38,405) mapped to 197 OTUs.

**Figure 1:**
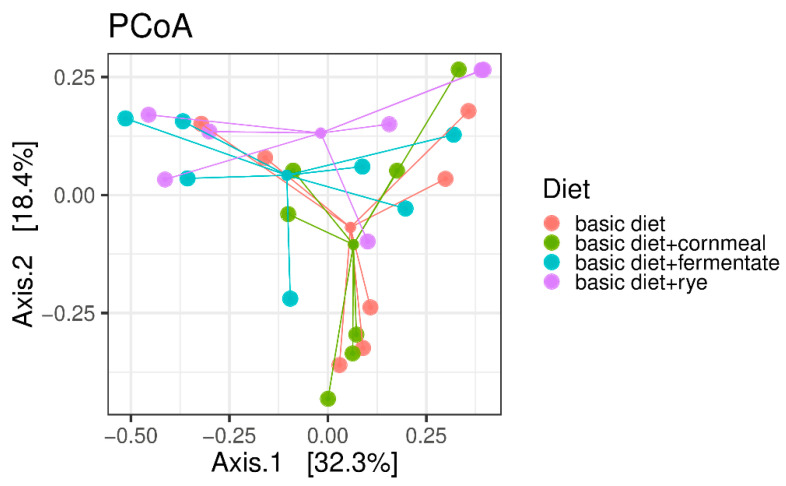
Bray–Curtis dissimilarity-based principal coordinate analysis (PCoA). Each point represents a different animal; coloured lines connect samples of one dietary treatment (vegetarian diet (basic diet) and the vegetarian diet supplemented with feather meal as well as either cornmeal (basic diet + cornmeal), fermented rye (basic diet + fermentate) or rye (basic diet + rye)).

The dietary treatment explained 11.6% of the sample’s variability but did not contribute significantly to the differences in microbial composition of the samples. Figure 1 shows the PCoA based on Bray–Curtis dissimilarity of samples.

**Figure 2:**
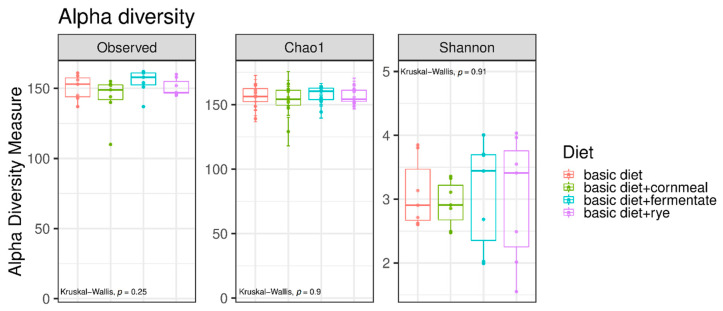
Alpha diversity in faecal samples of dogs. Box plots showing alpha diversity in samples using the species richness estimators Observed Species, Chao1 and Shannon index. Comparisons of the species richness estimators were done using the Kruskal–Wallis test (vegetarian diet (basic diet) and the vegetarian diet supplemented with feather meal as well as either cornmeal (basic diet + cornmeal), fermented rye (basic diet + fermentate) or rye (basic diet + rye)).

Comparisons of the measured species richness estimators—Observed Species, Chao 1 and Shannon index—revealed no statistically significant differences between the dietary treatments (Figure 2).

**Figure 3:**
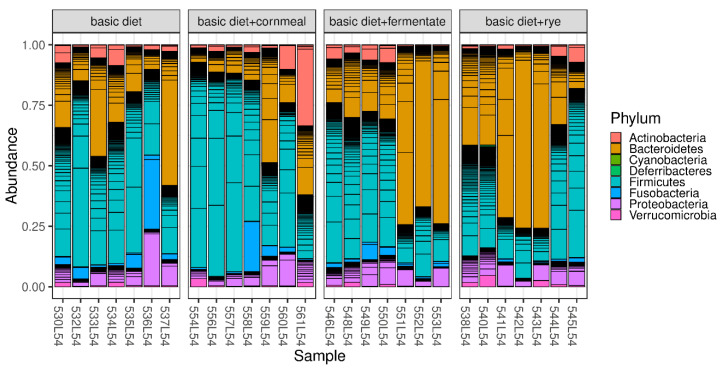
Bar charts represent the relative abundance of bacterial phyla determined using 16S rRNA gene sequencing in faecal samples of dogs offered different diets (vegetarian diet (basic diet) and the vegetarian diet supplemented with feather meal as well as either cornmeal (basic diet + cornmeal), fermented rye (basic diet + fermentate) or rye (basic diet + rye)).

Faecal microbiota were dominated by *Firmicutes* and *Bacteroidetes*. Relative abundances of bacterial phyla per sample are shown in Figure 3 separated by dietary treatment. The corresponding values averaged over the dietary treatments are shown in Table 3.

Multiple testing on normalized counts on each phylum and family yielded no significant differences between the dietary treatments. 

At the species level, no OTUs with significant different abundance (plus an additional log fold change criterion of ± 2) between dietary treatments were found.

**Figure 4:**
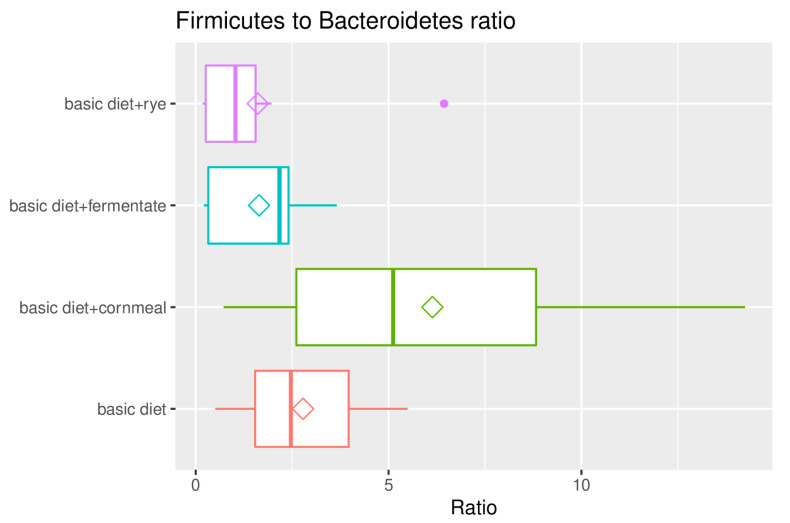
Box-and-whisker plot illustrating the *Firmicutes*/*Bacteroidetes* ratio in faecal samples of dogs fed the vegetarian diet (basic diet) or the vegetarian diet supplemented with feather meal as well as either cornmeal (basic diet + cornmeal), fermented rye (basic diet + fermentate) or rye (basic diet + rye). Group means are plotted additionally as rhombus while outliers are plotted individually as points.

The ratio of *Firmicutes* to *Bacteroidetes* in samples belonging to the different feeding groups were calculated in every single sample and plotted in groups as a box-and-whisker plot in Figure 4.

## 4. Discussion

The phyla *Bacteroidetes* and *Firmicutes* were the dominant microorganisms in the intestines of dogs in this study as in many other studies [36]. Even if the dietary treatment did not contribute significantly to the differences in microbial composition of the samples and multiple testing on each phylum yielded no significant differences between each dietary treatment and the basic diet in the present study, tendencies concerning the *Firmicutes* to *Bacteroidetes* ratio could be observed. The *Firmicutes* to *Bacteroidetes* ratio shifted to the advantage of *Firmicutes* when adding feather meal to the former vegetarian diet (basic diet compared to basic diet + cornmeal). It is known that the phylum of the mostly gram-positive *Firmicutes* occur more frequently in protein-rich diets and in obese individuals (both humans and dogs), which also shifts the ratio of *Bacteroidetes* to *Firmicutes* to the disadvantage of the *Bacteroidetes* [1,37,38,39]. The diets of the present study contained similar protein contents. The addition of cornmeal did not seem to compensate for this effect, while the addition of rye, fermented or not, shifted the *Firmicutes* to *Bacteroidetes* ratio back to the advantage of *Bacteroidetes*. With the addition of rye and fermented rye to the former vegetarian diet supplemented with feather meal, *Bacteroidetes* became the dominant phylum. It has to be taken into account that the analysis of chemical composition of the diets revealed differences in phosphor and calcium content. The phosphor and calcium content of the three non-vegetarian diets were lower compared to the vegetarian diet. Both minerals play an important role for intestinal microbiota [40,41]; phosphor is essential for bacterial degradation of dietary fibre, as their fibrolytic enzymes strongly depend on the supply of available phosphor, and Ca-phosphate has buffering functions in intestinal digesta [41]. Still, the shifts mentioned above were observed when rye was added. The gram negative *Bacteroidetes* are found in higher amounts in the intestine, especially in a carbohydrate/fibre-rich diet [39]. They produce short-chain fatty acids from the carbohydrates provided to them, with butyric acid/butyrate being the most favored end product [42]. In addition, bacteria of the genus *Bacteroidetes* interact with the intestinal immune system and with intraepithelial lymphocytes (increase of IL-6 secretion), thus leading to a strengthening of the intestinal barrier function [43]. Likewise, the promotion of normal intestinal development or the intestinal immune system and the associated reduction of colonization with pathogens should not remain unmentioned [44,45]. *Bacteroidetes* are also believed to have a detoxification function [46].

In rye, arabinoxylans, together with fructans and β-glucans, represent the main part of dietary fibre which cannot be broken down by the host’s own enzymes [20]. Thus, they reach the large intestine mostly intact and can be fermented there by bacteria, whereby, especially the fructans and arabinoxylans, which are more present in rye than in other cereals, stimulate butyrate formation [47,48,49]. In turn, numerous positive/health-promoting properties have been described for dietary fibres and the butyrate formed from them. There is a great deal of scientific interest in these substances, as the numerous publications in this field clearly show. A diet rich in dietary fibres/butyrate can reduce diseases like obesity, diabetes, cardiovascular diseases, gastrointestinal imbalances and also some cancer diseases. In addition, inflammatory processes can also be alleviated by butyrate such that a faster recovery can occur [49,50,51,52,53,54,55,56,57,58,59,60,61,62,63,64,65,66]. With regard to the bacterial colonization when rye and fermented rye were added, there were hardly any differences in faeces, so it can be stated that the fibre components important for the colon flora remain relatively untouched by a preceding fermentation. However, with regard to the different parts of the gastrointestinal tract, fermentation might have had influences on the small intestine’s microbiota, which can no longer be seen in faeces. When offered a non-fermented liquid feed compared to a fully fermented liquid feed to pigs, significant effects on the pig microbiota of the small intestine were seen in the experiments of Bunte et al. [23], while colonic and faecal microbiota seemed to remain unaffected.

Even though it was not statistically significant, the Shannon index in the samples of dogs fed a rye containing diet, fermented or not, was numerically higher compared with dogs offered the vegetarian as well as the former vegetarian diet supplemented with feather meal and cornmeal, while Observed Species and Chao 1 indices remained similar between the groups. In contrast to the measured species richness estimators Observed Species and Chao 1 index, the Shannon index characterizes species diversity accounting for the abundance and evenness of the species. This indicates that the faeces hosted a similar number of different bacterial species, but with the offer of rye-rich diets, the individuals among these bacterial species seemed to be more evenly distributed. The majority of studies of biodiversity–stability have predominantly examined species richness, but when microbial communities are highly uneven, or an extreme dominance by one or a few species is observed, their functioning is less resistant to environmental stress [67].

## 5. Conclusions

No statistically significant difference were found with regard to microbiota composition in dog faeces. Nevertheless, the *Firmicutes*/*Bacteroidetes* ratio seem to be affected by dietary treatment. The ratio tended to shift to the advantage of *Firmicutes* when feather meal and cornmeal were added and tended to switch back to the advantage of *Bacteroidetes* if rye was added. The results of the present preliminary study indicate that the addition of rye might have the potential to compensate possible unfavourable effects of diets with animal proteins on intestinal microbiota of dogs and further studies in this field are desirable.

## Figures and Tables

**Table 1:** Components of the vegetarian diet (basic diet) and the vegetarian diet supplemented with feather meal as well as either cornmeal (basic diet + cornmeal), fermented rye (basic diet + fermentate) or rye (basic diet + rye).

Components (% as Fed)	Basic Diet (Vegetarian)	Basic Diet + Cornmeal (non-Vegetarian)	Basic Diet + Fermentate (non-Vegetarian)	Basic Diet + Rye (non-Vegetarian)
Wheat	17.3	10.7	10.7	10.7
Broken rice	17.3	10.7	10.7	10.7
Wheat gluten	5.2	3.3	3.3	3.3
Rice protein	5.2	3.3	3.3	3.3
Sunflower oil	4.1	2.5	2.5	2.5
Dried beet pulp	1.8	1.1	1.1	1.1
Brewer’s yeast	1.2	0.7	0.7	0.7
Linseed	1.2	0.7	0.7	0.7
Lignocellulose	0.5	0.3	0.3	0.3
Sum of additives ^1^	5.7	3.5	3.5	3.5
Feather meal	-	2.7	2.7	2.7
Cornmeal	-	20.1	-	-
Rye	-	-	-	20.1
Fermented rye	-	-	60.4 ^2^	
Water	40.5	40.3	-	40.3

^1^ dicalcium phosphate, sodium chloride, potassium chloride, choline chloride, L-lysine, DL-methionine, magnesium carbonate, taurine, protein hydrolysates with additives such as synthetic amino acids, sugars and phosphoric acids and antioxidants; ^2^ dry matter content: 30.7%. Percentages may not total to 100% due to rounding.

**Table 2:** Chemical composition (in g/kg dry matter if not other stated) of the vegetarian diet (basic diet) and the vegetarian diet supplemented with feather meal as well as either cornmeal (basic diet + cornmeal), fermented rye (basic diet + fermentate) or rye (basic diet + rye).

	Basic Diet (Vegetarian)	Basic Diet + Cornmeal (non-Vegetarian)	Basic Diet + Fermentate (non-Vegetarian)	Basic Diet + Rye (non-Vegetarian)
Moisture (% as fed)	44.9	39.7	41.3	42.7
Crude ash	53.8	42.6	41.8	41.3
Crude fat	67.5	72.7	71.2	62.9
Crude protein	220	218	214	214
Crude fibre	18.3	21.6	18.0	17.0
N-free Extracts	640	645	655	665
Starch	468	435	483	482
Sugar	26.3	42.2	39.9	48.0
ME (MJ/100 g as fed) ^1^	0.89	0.98	0.96	0.93
Calcium	9.92	6.85	7.00	6.86
Phosphorus	3.86	3.01	3.09	2.99
Lysine	10.0	7.41	8.02	8.22
Methionine	6.13	4.29	4.35	4.45
Threonine	6.33	6.34	6.60	6.76
Tryptophan	2.25	1.74	1.93	1.98

^1^ Metabolizable energy (ME) content of the diets was estimated in accordance with the NRC [29]. Amino acid contents were calculated. Sums of crude ash, crude fat, crude protein, crude fibre and N-free extracts may not total to 1000 g due to rounding.

**Table 3:** Relative abundance (mean ± s.d., in %) of bacterial phyla > 1% and *Firmicutes/Bacteroidetes* ratio (*F*/*B* ratio, median) in faecal samples of dogs fed the vegetarian diet (basic diet) or the vegetarian diet supplemented with feather meal as well as either cornmeal (basic diet + cornmeal), fermented rye (basic diet + fermentate) or rye (basic diet + rye).

Phylum	Basic Diet (Vegetarian)	Basic Diet + Cornmeal (non-Vegetarian)	Basic Diet + Fermentate (non-Vegetarian)	Basic Diet + Rye (non-Vegetarian)	adj. *p*-Value ^1^
*Actinobacteria*	3.94 ± 3.16	7.56 ± 11.8	3.49 ± 2.97	2.48 ± 2.54	0.771
*Bacteroidetes*	26.7 ± 16.4	18.0 ± 14.8	43.2 ± 26.3	48.5 ± 25.2	0.378
*Firmicutes*	51.7 ± 17.1	61.3 ± 24.2	41.5 ± 21.0	37.7 ± 21.3	0.698
*Fusobacteria*	7.08 ± 10.5	4.24 ± 7.44	2.64 ± 2.20	0.48 ± 0.66	0.771
*Proteobacteria*	9.50 ± 6.58	7.76 ± 4.18	8.37 ± 2.86	8.81 ± 2.82	0.922
*Verrucomicrobia*	0.82 ± 0.72	0.98 ± 1.19	0.62 ± 0.63	1.57 ± 1.68	0.771
*F*/*B* ratio	2.47	5.12	2.17	1.03	

^1^*p*-values were adjusted by the Benjamini and Hochberg (BH) method to control for the false discovery rate (FDR) of 5%.
